# A Novel Homozygous 9385 bp Deletion in the *FERMT1* (*KIND1*) Gene in a Malaysian Family with *Kindler Epidermolysis bullosa* and a Review of Large Deletions

**DOI:** 10.3390/ijms26094237

**Published:** 2025-04-29

**Authors:** Alfred Klausegger, Fabian Leditzky, Susanne Krämer, Francis Palisson, María Joao Yubero, Sebastián Véliz, Mark Jean Aan Koh, Ene-Choo Tan, Martin Laimer, Johann Wolfgang Bauer, Ignacia Fuentes

**Affiliations:** 1 EB House Austria, Department of Dermatology and Allergology, University Hospital of the Paracelsus Medical University, 5020 Salzburg, Austria; fabian.leditzky.pm@protonmail.ch (F.L.); m.laimer@salk.at (M.L.); joh.bauer@salk.at (J.W.B.); 2 Special Care Clinic, Universidad de Chile, Olivos 943, Independencia, Santiago 8380544, Chile; susiks@yahoo.com (S.K.); sveliz@odontologia.uchile.cl (S.V.); 3 Servicio de Dermatología, Facultad de Medicina Clínica Alemana, Universidad del Desarrollo, Santiago 7591538, Chile; francis.palisson@gmail.com; 4 DEBRA Chile, Santiago 8580670, Chile; mjyubero@debrachile.cl; 5 Pediatrics and Pediatric Infectious Diseases of Clínica Alemana, Facultad de Medicina Alemana, Universidad del Desarrollo, Santiago 7591538, Chile; 6 Department of Dermatology, KK Women’s and Children’s Hospital, Singapore 229899, Singapore; mark.koh.j.a@singhealth.com.sg; 7 Research Center, KK Women’s and Children’s Hospital, Singapore 229899, Singapore; tan.ene.choo@kkh.com.sg; 8 Department of Dermatology and Allergology, University Hospital of the Paracelsus Medical University, 5020 Salzburg, Austria; 9 Centro de Genética Y Genómica, Facultad de Medicina Clínica Alemana, Universidad de Desarrollo, Santiago 7610658, Chile; 10 Facultad de Ciencias Biológicas, Pontificia Universidad Católica de Chile, Santiago 8320165, Chile

**Keywords:** Kindler Epidermolysis bullosa, *FERMT1*, poikiloderma, deletion, amelogenesis imperfecta

## Abstract

Kindler Epidermolysis bullosa (KEB; OMIM 173650) is a rare autosomal recessive genodermatosis characterized by bullous poikiloderma and photosensitivity. Additional presentations include blistering, poor wound healing, skin atrophy, and increased risk of skin cancer. Most cases of KEB result from aberrations in the *FERMT1* (Fermitin family member 1) gene encoding kindlin-1 and include nonsense, frameshift, splicing, and missense variants. Large deletion variants have been reported in nine cases to date. Most variants are predicted to lead to premature termination of translation and to loss of kindlin-1 function. In this study, we report on a 33-year-old male patient who presented with typical clinical manifestations of KEB. As routine molecular testing failed to obtain a diagnosis, Next Generation Sequencing (NGS) of an Epidermolysis Bullosa (EB)-specific panel was carried out followed by the determination of the deletion breakpoints and verification at the mRNA and protein levels. This approach revealed a new large homozygous deletion of ~9.4 kb in the *FERMT1* gene involving exons 7 to 9. Finally, we performed a literature review on large *FERMT1* deletions. The deletion is predicted to skip exons 7 to 9 within the mRNA, which results in a frameshift. The patient’s phenotype is likely caused by the resulting truncated and non-functioning protein. Our report further enriches the spectrum of *FERMT1* gene variants to improve genotype–phenotype correlations.

## 1. Introduction

Kindler Epidermolysis bullosa (KEB; formerly Kindler Syndrome, OMIM 173650) is an autosomal recessive mechanobullous genodermatosis, which was first described by Theresa Kindler in 1954 [1,2]. It is caused by pathogenic loss-of-function variants in the *FERMT1* gene encoding a focal adhesion protein, which is predominantly expressed in skin basal keratinocytes, periodontal tissues, and colon. Kindlin-1 plays a critical role in linking the actin cytoskeleton with the underlying extracellular matrix anchorage network.

Clinically, it presents with acral blistering that manifests at birth or in early infancy. In addition, patients suffer from photosensitivity, which is severe during childhood and usually improves after adolescence. Further clinical symptoms include progressive poikiloderma, cutaneous atrophy, palmoplantar hyperkeratosis, and pseudosyndactyly. Moreover, gingival fragility and oesophageal and gastrointestinal involvement is observed [3,4,5]. Diagnostic algorithms based on clinical symptoms have been proposed by Angelova-Fischer (2005) and Chopkar (2021) [3,6]. Ultrastructural hallmarks of KEB include keratinocyte separation, reduplication in the lamina densa, and the formation of fissures at different rates and levels of the dermoepidermal structure [7,8].

The treatment of KEB is mainly symptomatic and relies on the prevention of skin trauma, proper wound care, the prevention of infections, and surgical correction of stenoses. Antioxidants and flavonoid luteolin may reduce UV-B-induced keratinocyte apoptosis [9].

Approximately 250 cases [6] and a total of at least 107 molecular variants have been reported in the HGMD Professional 2023.4 (https://www.hgmd.cf.ac.uk/ac/gene.php?gene=FERMT1), accessed on 2 April 2025), which comprise nonsense and splicing variants as well as small and large deletions and insertions.

In this study, we present a 33-year-old male patient from Malaysia, with clinical signs of KEB. While no pathogenic variants in *FERMT1* or any other EB-related gene could be found by exome sequencing, advanced molecular profiling identified a novel homozygous 9385 bp deletion variant spanning exons 7–9 in the *FERMT1* gene. These results were correlated with the literature data on large *FERMT1* deletions.

## 2. Results

### 2.1. The Patient

**Figure 1:**
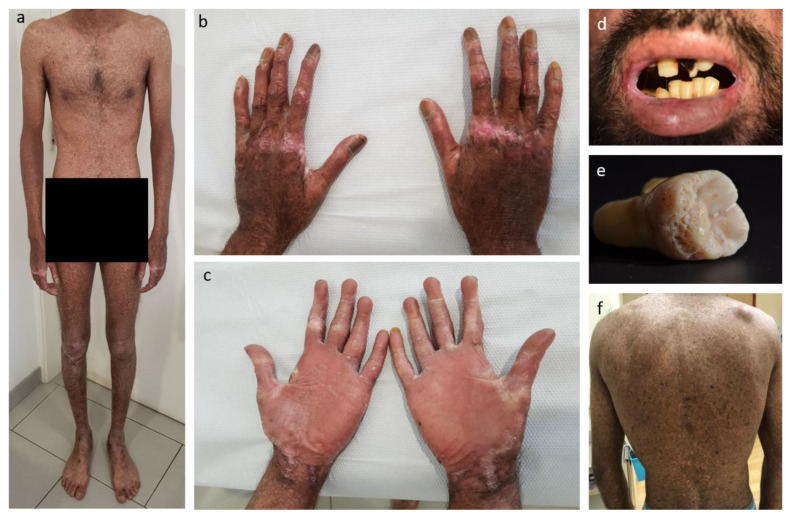
Clinical features of patient. (**a**,**b**,**f**) Poikiloderma; (**b**) nails dystrophy with brownish discoloration and parallel lines of nail plate; (**c**) proximal pseudosyndactyly and absence of linearity in palms; (**d**) patient had undergone oral reconstruction due to severe microstomia with maximal interincisal opening of 6 mm; angular cheilitis; severe caries and lower lip leukoplakia. (**e**) early teeth extraction showing hypoplastic pitted syndromic amelogenesis imperfecta with generalized pits.

The index patient presented with chronic generalized skin blistering and wounding starting from birth, microstomia (Figure 1d), pseudosyndactyly, palmoplantar keratoderma, poikiloderma, diffuse hypo- and hyperpigmentation, and nail atrophy (Figure 1a–c,f). He further complained about recurrent skin infections and severe constipation. Oral examination showed severe microstomia with a maximal interincisal opening of 6 mm. The patient also presented with cheilitis, vestibular obliteration, periodontal disease, angular cheilitis, recurrent intraoral lesions and blisters, pitting enamel hypoplasia, multiple caries, and lower lip leukoplakia (Figure 1d).

Family history revealed that the parents of the index patient were first cousins without any history for skin diseases. Likewise, their older son was reported as clinically asymptomatic.

Later in his evolution, the patient also developed urethral stenosis, which had to be operated on.

Interestingly, he also exhibits amelogenesis imperfecta (Figure 1e), which has not been reported for KEB but frequently found in another type of EB, Junctional EB (JEB). Thus, the clinical phenotype was suggestive of a skin fragility disorder, particularly of Junctional or Kindler EB.

Our patient was initially treated and followed in Singapore, where a clinical exome had been performed to determine the molecular cause of his disease. At this point, no clear pathogenic EB-related variants had been found but only heterozygous single nucleotide polymorphisms in recessive EB-causing genes.

### 2.2. NGS-Based Variant Analysis

NGS-based data analysis with the Ion Reporter software (Version 5.20, Life Technologies, Carlsbad, CA, USA) revealed no pathogenic variant in the *FERMT1* (NM_017671.5) nor in any of the other genes of the Epidermolysis bullosa panel. While three *LAMB3* heterozygous missense variants (M852L, A926D, N690S) were detected, these variants were considered to be non-pathogenic, referring to the considerably high minor allele frequency in the normal population.

**Figure 2:**
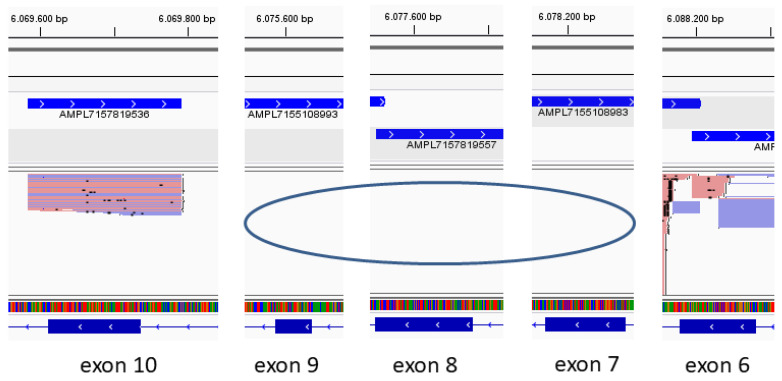
IGV illustration on EB Panel Next Generation Sequencing. Exonic copy number analysis revealed absence of sequencing reads for exons 7, 8, and 9.

Integrative Genome Viewer (IGV, https://software.broadinstitute.org/software/igv/) [10], accessed on 24 November 2022, analyses revealed that amplicons from exons 7, 8, and 9 of the patient’s allele have not been amplified (Figure 2).

### 2.3. Variant Identification and Sequence Analysis

To determine the 5′ and 3′ breakpoints of the deletion, a series of successive polymerase chain reactions (PCR) were performed within ~10 kb of intron 6 and ~5.8 kb of intron 9. The primer combination pr6F6 5′ACAATGCTTTACAGTGGCAAA3′ and pr9R2 5′GCCAAATATGAGGGCAATTTT3′ located nearby the supposed two breakpoints were designed to amplify a fragment of 894 bp in the case of the deleted allele of the homozygous patient and the heterozygous parents and helped us to confirm the deleted sequences by Sanger sequencing.

**Figure 3:**
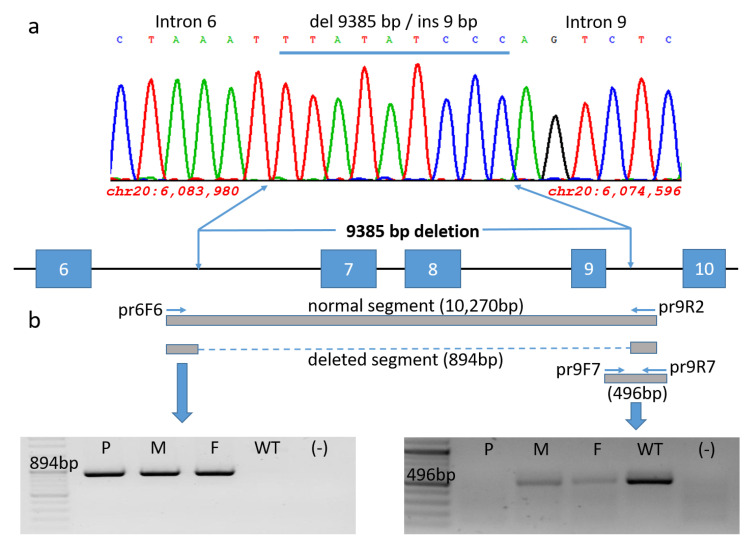
A schematic illustration of the 9385 bp deletion in the *FERMT1* genomic region spanning exons 7–9: (**a**) The Sanger sequence of the patient’s 894 bp fragment revealed 5′ and 3′ breakpoints (chr20:6,083,980; chr20:6,074,596, GRCh37/hg19) within intron 6 and 9, thereby confirming the 9 kb deletion with an 9 bp insertion instead (TTATATCCC). (**b**) Verification of the deletion by two different polymerase chain reactions. The primer combination pr6F6 and pr9R2 (both located outside of the deletion) was used to amplify the region spanning the breakpoints. Its application resulted in a fragment of 894 bp in the homozygous patient and heterozygous parents, reflecting the deleted allele. A dashed line is delineating the indel region. A single band of 894 bp was observed in the homozygous patient (P) and his heterozygous mother (M) and father (F), whereas the wildtype sample (WT) without deletion is lacking the band (left panel). A 496 bp fragment using primer pr9F7 (located within the deletion region) and pr9R7 (located outside) was observed only in the mother (M), father (F), and the wildtype sample (WT). An amplification within the patient’s deletion region was not found.

Profiling the 5′ and 3′ breakpoints (chr20:6,083,980; chr20:6,074,596; GRCh37/hg19) within intron 6 and 9 confirmed the 9 kb deletion and showed an insertion of 9 bp instead (TTATATCCC) (Figure 3a,b).

The deletion was further confirmed by using a primer combination of primer pr9F7 5′AACGGGCACTTCACTGGG3′ complementary to the deletion and primer pr9R7 5′AGCACTTTGGGAGGCCAAG3′ located outside the deletion. This application resulted in a 496 bp fragment observed only in both heterozygous parents and the wildtype sample (Figure 3b).

### 2.4. Genetic Analysis on mRNA Level

**Figure 4:**
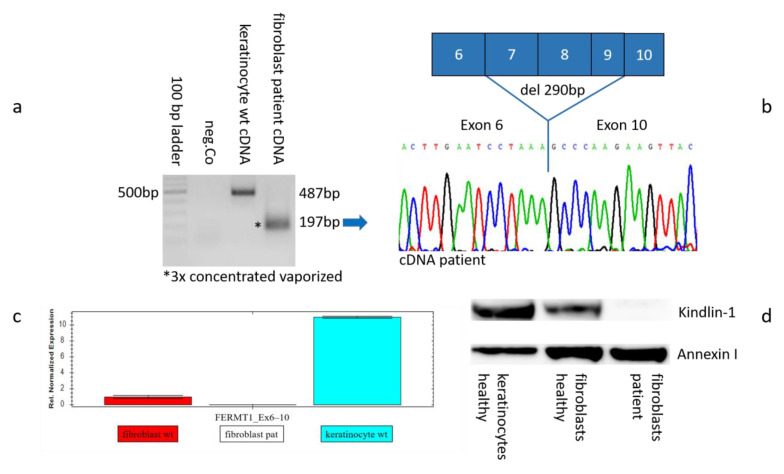
Verification of the 9385 bp deletion and variant consequences at mRNA and protein levels. (**a**) RT-PCR analysis of total RNA from patient fibroblasts resulted in a unique PCR product of 197 bp, which was threefold concentrated and vaporized before gel loading. Amplification from wildtype keratinocytes revealed the normal product of 487 bp. Mw, 100 bp DNA ladder. (**b**) The 197 bp PCR product was sequenced by Sanger and corresponded to an out-of-frame transcript (del 290 bp) carrying the deletion of exons 7, 8, and 9. (**c**) Measurement by quantitative real-time PCR. qPCR analysis was performed using cDNA derived from patient’s fibroblasts, as well as wildtype control fibroblast and keratinocyte cell culture. RT-Primers were located within the sequence of exons 6 and 10 surrounding the deletion (for primer sequences refer to 4.5). The results are depicted as relative expression levels set to a value of 1 for wildtype fibroblasts and normalized to the GAPDH gene +/− standard error. Patient’s mRNA from fibroblasts did not show any amplification. Wildtype mRNA from keratinocytes resulted in a ~10× higher expression compared to wildtype mRNA from fibroblasts. (**d**) Western blot analysis. Incubation with an antibody directed against kindlin-1 indicates complete loss of kindlin protein in patient’s fibroblasts. The healthy keratinocytes show a higher expression level compared to the control fibroblasts. Antibody directed to Annexin I served as the loading control.

Reverse transcription-PCR amplification of the total RNA extracted from primary fibroblast cell culture from the patient and a keratinocyte cell culture from an unaffected individual (healthy control) resulted in a unique PCR product of 197 bp compared to the 487 bp control band on agarose gel. In order to obtain sufficient product for sequencing, the PCR product of 197 bp was threefold concentrated and vaporized before gel loading and sequencing to attenuate the effects of mRNA decay and strongly reduced expression of truncated RNA molecules (Figure 4a).

Sanger sequencing of the 197 bp PCR product obtained from the cDNA of the patient showed that it was missing 290 bp compared to the control sequence. This observation corresponds to the out-of-frame transcript carrying the deletion of exons 7, 8, and 9 (Figure 4b).

### 2.5. Gene Expression on RNA and Protein Levels

To assess the consequences of the 9 kb deletion, we studied *FERMT1* expression with quantitative real-time PCR analysis performed on the cDNA derived from the fibroblast and keratinocyte cell culture established from the patient and control skin, respectively. RT-Primers were designed to target the sequence within exons 6 and 10, encompassing the deletion. As expected, no amplification was observed on the patient’s fibroblasts. Wildtype mRNA from keratinocytes resulted in extensively higher expression compared to wildtype mRNA from fibroblasts (Figure 4c).

Immunoblot analysis revealed complete loss of kindlin protein in the fibroblasts of the patient. In contrast, regular staining intensity of anti-kindlin antibody in the control keratinocytes compared to the healthy fibroblasts indicated 2.5× higher density in densitometric blot analysis (Figure 4d).

## 3. Discussion

In 2003, the genetic defect of KEB was first localized to loss-of-function variants in the *FERMT1* (also formally named *KIND1*) gene mapped to chromosome 20p12.3 [7]. It codes for kindlin-1 protein, a 677-amino acid protein, expressed particularly in the basal keratinocytes, intestine, and kidney, whose loss results in defects in actin–extracellular matrix linkages and abnormal skin fragility [11].

In this paper, we report on a 33-year-old Malaysian patient with clinical findings suggestive of KEB. While the application of clinical exome and Ion Reporter software was unable to reveal any underlying molecular aberration, advanced molecular profiling using IGV (Integrative Genome Viewer) [10] revealed a previously unreported/new large *FERMT1* homozygous deletion spanning exons 7–9. This deletion was later confirmed to be inherited from his parents who are both heterozygous carriers.

In more detail, we designed a primer pair targeting the breakpoint proximity of the 3017 bp deletion published by Zhou (2009) [12] to amplify a 400 bp fragment in the case of the deletion and 3417 bp in the case of the healthy control. We failed to amplify the 400 bp fragment that implicated an extended deletion far outside of this fragment.

Consequently, a series of successive polymerase chain reactions (PCR) were performed within ~10 kb of intron 6 and ~5.8 kb of intron 9 to explore the 5′ and 3′ breakpoints of the deletion which could be estimated within intron 6 and 9 (chr20:6,083,980; chr20:6,074,596; GRCh37/hg19) and herewith confirmed a 9 kb deletion.

An insertion of 9 bp (TTATATCCC) was detected between the recombination sites (Figure 3a). The primer combination pr6F6-ACAATGCTTTACAGTGGCAAA and pr9R2-GCCAAATATGAGGGCAATTTT designed in proximity of the two breakpoints amplified a fragment of 894 bp in the case of the deleted allele of the homozygous patient. This primer pair may serve as a PCR-based diagnostic substrate for rapid screening for this specific deletion in KEB patients.

The 9 kb deletion was also verified on mRNA and protein levels. RT-PCR analysis of the cultured patient fibroblasts performed in a single PCR product of 197 bp. “PCR end point” analysis resulted in minimized amplification levels presumably due to mRNA decay. Nonsense-mediated mRNA decay might downregulate the mature mRNA, a unique process shown for most of the variants identified in the *FERMT1* gene [13,14]. Therefore, it was required to concentrate the volume of PCR by vaporization for gel loading and sequencing. Sanger sequencing verified out-of-frame transcription (del 290 bp) and deletion of exons 7, 8, and 9 (Figure 4a,b).

This result is also consistent with the measurement by quantitative real-time PCR. We demonstrated that mRNA from patient fibroblasts lack measurable amplification, which most likely reflects the effect of mRNA decay. Noteworthy, mRNA from wildtype keratinocytes resulted in a significantly higher expression (~10-fold) compared to wildtype fibroblasts (Figure 4c). The results of the immunoblot analysis corroborate the results at the mRNA level. Complete loss of kindlin-1 protein was shown by an antibody directed against kindlin-1 in the patient fibroblasts. The healthy keratinocytes show densely higher expression level compared to the control fibroblasts, as the densitometric analysis of the blot calculated a value of ~2.5-fold higher (Figure 4d).

Our data indicate that the deletion leads to the generation of a truncated protein lacking a substantial part of its c-terminal end. This aberration is predicted to cause mRNA decay. However, a reduced amount of c-terminally truncated kindlin-1 in keratinocytes cannot function correctly [15].

The nine *FERMT1* deletions hitherto described in the literature are predicted to result in complete loss of full-length kindlin-1 protein and diminished function [12,13,16,17,18,19,20,21,22].

- Most of them are out-of-frame deletions and affect one or more exons.
- Only one deletion, affecting exons 2–6, is reported to be in-frame with the annotation that the deletion range also contains the translation initiation site (TIS) ATG located in exon 2 [17].
- A deletion eliminating exons 14 and 15 extended beyond the *FERMT1* coding region and 3′UTR, leading to partial mRNA decay [16].
- A large deletion (g.-711-1241del), that spanned the putative *FERMT1* promoter sequence and the first noncoding exon of the gene including the predicted transcription start site, was associated with a complete blockade and prevention of *FERMT1* transcription [18].

DNA double-stand breaks (DSBs) can be caused by endogenous and exogenous DNA damaging agents. Two major pathways are responsible for the repair of DSBs, i.e., homologous recombination and nonhomologous end-joining. If the repair is failing, these DSBs may result in chromosomal re-organization [23].

Blasting the insertion sequence “TTATATCCC” occurring between the two recombination sites resulted in a homologous sequence 7228 bp upstream, whereas sequencing of this region confirmed no conspicuousness.

Misaligned repetitive elements (e.g., Alu sequences) often serve as a mediator for homologous recombination [23,24,25]. Has (2006) calculated an abundance of 52% of all noncoding regions of *FERMT1* as repetitive sequences. Almost half of these repeats represented Alu elements [13].

An analysis of intronic intersperced repeat elements around the breakpoints by the RepeatMasker software (http://www.repeatmasker.org/cgi-bin/WEBRepeatMasker), accessed on 5 November 2023, resulted in the matching repeats L1ME4b of the LINE/L1 family and AluSx of the SINE/Alu family at the 5′ and 3′ breakpoints, respectively. The homologous recombination of the deletion breakpoints embedded in these repeats could be mediated by a combination of these elements.

Zhou (2009) [12] reported a similar but different deletion of 3017 bp including exons 7–9. However, these authors assumed matrix attachment regions (MARs), rather than being mediated by Alu elements to cause homologous recombination. The short homology sequence “GA” across the deletion junction, the sequence “TTTAAA” near the 3′ breakpoint, and the MARs near the 5′ and 3′ breakpoints favor involvement in nonhomologous end-joining [12]. The homology region between the two breakpoints was not demonstrated in this case, instead, a 9 bp insertion occurs but linkage to the homologous sequence upstream does not seem to exist.

Most *FERMT1* variants comprising nonsense, splice site, small indel, and missense variants can be detected by NGS EB panel sequencing and Sanger sequencing, which both represent the current diagnostic standard. However, in distinct cases, direct sequencing of the coding region of the *FERMT1* gene may fail to reveal the underlying variant, which may partly also account for the rather limited number of published cases with large scale deletion (Table 1) [13,26]. Therefore, advanced screening approaches for *FERMT1* gene variants must allow for the evaluation for large deletions. In addition, the molecular assessment of patients implicated to suffer from KEB includes other genes that cause KEB-like features [27,28,29]. For example, a homozygous sequence variant was identified at the +4 position of intron 2 in the *USB1* gene, encoding an exoribonuclease required for processing of U6 snRNA, a critical component of spliceosomes and another homozygous donor splice site mutation involving exon/intron 5 of tetraspanin *CD151*, a component of hemidesmosomes, as well as a key player in wound healing and the re-epithelialization process [30,31], were identified to cause KEB-like phenotypes [32].

## 4. Materials and Methods

### 4.1. Study Approval

Informed written consent was obtained from the patient and his parents prior to sample collection. This study was performed in accordance with the Declaration of Helsinki and approved by the Ethics committee from Clínica Alemana Universidad del Desarrollo # 2017-85.

### 4.2. NGS-Based Variant Analysis

Genomic DNA was isolated from the peripheral blood of the patient and his mother and the saliva of the patient’s father. Commercial kits were used to isolate DNA from the samples. An EB-specific sequencing panel including the *FERMT1* gene was used. Sequencing was performed on 10 ng of DNA from the patient with a Personal Genome Machine (PGM) from Life Technologies according to the protocol of the company and analyzed with the Ion Reporter Software (Life Technologies, Carlsbad, CA, USA).

### 4.3. Primary Cell Isolation

A 4 mm punch biopsy was taken from uninjured skin from the patient’s lower back. The biopsy was collected in saline solution and taken immediately to the laboratory for processing. Primary fibroblasts of the patient’s skin and normal human skin were cultured at 37 °C with 5% CO_2_, in DMEM (catalog no. SH30243.02; Cytiva/Hyclone, Emerville, CA, USA), which was supplemented with 10% fetal bovine serum (catalog no. F2442; Sigma-Aldrich, Darmstadt, Germany).

Primary keratinocytes of normal human skin were cultured at 37 °C with 5% CO_2_, in Epilife medium supplemented with HKGS and calcium chloride (catalog no. MEPICF500; Cascade, Portland, OR, USA).

### 4.4. PCR and Sanger Sequencing

All primers used for sequential PCR were designed by freeware Primer3 (v. 0.4.0) [https://bioinfo.ut.ee/primer3-0.4.0/], accessed on 5 March 2023. PCR was performed by GoTaq enzyme denaturing at 94 °C for 2min, followed by 35 cycles at 94 °C for 30 s, 57 °C for 45 s, and 72 °C for 60 s. The reaction ended with incubation at 72 °C for 5 min (Promega, Fitchburg, MA, USA). The PCR products were purified and direct sequencing of the fragments was carried out with an ABI 3500 automatic sequencer (Applied Biosystems, Waltham, MA, USA).

### 4.5. Real-Time PCR

Quantitative PCR analysis was performed using cDNAs derived from the patient’s fibroblast, as well as wildtype control fibroblast and keratinocyte cell culture. Total RNA was isolated using the RNeasy Mini kit (Qiagen, Hilden, Germany). RNA was incubated with DNaseI (Sigma Aldrich, St. Louis, MO, USA) and transcribed into cDNA with the iScript cDNA Synthesis Kit (BioRad, Hercules, CA, USA) according to the manufacturer’s protocol. Real-time PCR was performed with the GoTaq qPCR Master Mix (Promega, Fitchburg, MA, USA) on the CFX96 Real-Time PCR Detection System (Bio-Rad, Hercules, CA, USA).

The primers used were *FERMT1*_6for 5′ACTCCTCACGCTCCCTTATG3′ and *FERMT1*_10rev 5′GGTTCTCCTTGTTCAAGTTCCT3′; *GAPDH*_8F 5′GCCAACGTGTCAGTGGTGGA3′ and *GAPDH*_9R 5′CACCACCCTGTTGCTGTAGCC3′ were used as the internal reference control. The thermocycling conditions included an initial step at 94 °C for 2 min, followed by 40 cycles at 94 °C for 30 s, 57 °C for 45 s, and 72 °C for 60 s, and the reaction ended with a melting curve.

### 4.6. Protein Isolation and Western Blot

Cells were lysed with a radioimmunoprecipitation assay (RIPA) buffer (Santa Cruz Biotechnology, Heidelberg, Germany) and centrifuged at 10,000 rpm for 5 min. Clear supernatant was further used and frozen at −20 °C. Proteins were mixed with 4× loading buffer (0.25 M tris, 8% SGS, 30% glycerol, 0.02% bromphenol blue, pH 6.8) before denaturation at 95 °C for 5 min. Western blot analysis was then performed as previously described [33]. Ponceau red staining (Sigma-Aldrich, St. Louis, MO, USA) was used after blotting to estimate the protein loading. Nitrocellulose membrane was blocked via blocking reagent (Roche Diagnostics GmbH, Mannheim, Germany) diluted 1:10 in Tris-buffered saline with 0.1% Tween (TBS-T) for 1 h at room temperature. Kindlin-1 was detected via a polyclonal antibody (#720123, Thermo Fisher Scientific, Rockford, IL, USA) at a dilution of 1:1000 in blocking reagent (diluted 1:20 in TBS-T). The membrane was incubated overnight at 4 °C with the first antibody. The antibody directed to Annexin I (catalog no. sc-12740; Santa Cruz Biotechnology, Heidelberg, Germany) diluted 1:1000 in blocking reagent (diluted 1:10 in TBS-T) was used as the internal reference. A goat anti-rabbit HRP-labeled antibody (Dako, Santa Clara, CA, USA) was used as the secondary antibody. The membrane was incubated for 1 h at a dilution of 1:5000 in TBS-T. Protein bands were visualized with the ECL Select Western blotting Detection Reagent (GE Healthcare, Chicago, IL, USA) and the ChemiDoc MP Imaging System (BioRad, Hercules, CA, USA). Densitometric analysis was performed using the software Image Lab (BioRad, Hercules, CA, USA).

## 5. Conclusions

Advanced molecular profiling including NGS exome and panel sequencing identified a novel homozygous 9385 bp deletion variant spanning exons 7–9 in the *FERMT1* gene that was confirmed by profiling the 5′ and 3′ breakpoints and further analyses on the mRNA and protein levels. To our knowledge, this is the first large deletion identified in Malaysian patients with KEB, joining a collection of various large deletions and expanding the variant database.

## Figures and Tables

**Table 1:** A comprehensive collection of the large deletions in *FERMT1* compiled from the literature. Diagnostic PCR for detecting the specific deletions including primer sequences is listed whenever made available in the literature.

Size	Primer Reverse	Primer Forward	Diagnostic PCR	Reference	Exon	Deletion	Variant	Geographic Origin	Consanguinity	Sex	Age	Patient
362 bp	GTGCATGTGTTGCTACGTGC	TGCCTGTAATCCCAGCTACC	intron 9/11	[13]	10–11	3919 bp	g.70250_74168del	Southern Italy (Calabria)	No	F	41	1
362 bp	GTGCATGTGTTGCTACGTGC	TGCCTGTAATCCCAGCTACC	intron 9/11	[13]	10–11	3919 bp	g.70250_74168del	Southern Italy (Calabria)	Yes	F	16	2
362 bp	GTGCATGTGTTGCTACGTGC	TGCCTGTAATCCCAGCTACC	intron 9/11	[13]	10–11	3919 bp	g.70250_74168del	Southern Italy (Calabria)	Yes	F	25	3
2 kb	GGCTGCCAATAATGTTGGTT	CTTGCACCAGCTACCCTCTC	intron 13/15	[16]	14–15	8241 bp	g.80929_89169del *	Southern Bulgaria	No	M	32	4
2 kb	GGCTGCCAATAATGTTGGTT	CTTGCACCAGCTACCCTCTC	intron 13/15	[16]	14–15	8241 bp	g.80929_89169del *	Southern Bulgaria	No	F	28	5
2 kb	GGCTGCCAATAATGTTGGTT	CTTGCACCAGCTACCCTCTC	intron 13/15	[16]	14–15	8241 bp	g.80929_89169del *	Southern Bulgaria	No	F	56	6
763 bp	ATGACAGAGCCCATTTCCTG	AGGCAAGCAGTTAGGCCTAAT	intron 1/6	[17]	2–6	17,251 bp	g.6121241_6103991del	China	Yes	F	7	7
425 bp	CTTGGCATTGAACTGTTCGA	GGGGACAGAACAAGACTCCA	promoter/intron 1	[18]	1_TSS ^2^	1952 bp	g.-711-1241del	Middle Eastern	Yes	F	n.a.	8
425 bp	CTTGGCATTGAACTGTTCGA	GGGGACAGAACAAGACTCCA	promoter/intron 1	[18]	1_TSS ^2^	1952 bp	g.-711-1241del	Middle Eastern	Yes	F	n.a.	9
425 bp	CTTGGCATTGAACTGTTCGA	GGGGACAGAACAAGACTCCA	promoter/intron 1	[18]	1_TSS ^2^	1952 bp	g.-711-1241del	Middle Eastern	Yes	M	n.a.	10
425 bp	CTTGGCATTGAACTGTTCGA	GGGGACAGAACAAGACTCCA	promoter/intron 1	[18]	1_TSS ^2^	1952 bp	g.-711-1241del	Middle Eastern	Yes	M	n.a.	11
2693 bp	GCTCTCCAGGGCATTACAAG	CAATGCCACAGAAAGCTGAA	promo1/intron 1	[19]	1_TSS ^2^	38,222 bp	g.6140393_6102171 delinsCAAACTGA	India	Yes	M	4	12
425 bp	CTTGGCATTGAACTGTTCGA	GGGGACAGAACAAGACTCCA	promoter/intron 1	[19]	1_TSS ^2^	1952 bp	g.-711-1241del	Southern Italia (West Sicily)	No	M	22	13
362 bp	GTGCATGTGTTGCTACGTGC	TGCCTGTAATCCCAGCTACC	intron 9/11	[19]	10–11	3919 bp	g.70250_74168del *	Southern Italia (West Sicily)	No	M	15	14
400 bp	AGCCCAAGGGGAGGTATATT ^3^	TGGCTCACGCCTGTAATTC ^3^	intron 6/9	[12]	7–8–9	3017 bp	g.63601_66617del	China	Yes	M	23	15
894 bp	GCCAAATATGAGGGCAATTTT	ACAATGCTTTACAGTGGCAAA	intron 6/9	this study	7–8–9	9385 bp	g.6083980_6074596 delinsTTATATCCC	Malaysia	Yes	M	33	16
n.a.	n.a.	n.a.	n.a.	[20]	5	2664 bp ^1^	g.6109607_6112272del	Iran	Yes	n.a.	n.a.	17
n.a.	n.a.	n.a.	n.a.	[21]	10	7055 bp	c.(1139+1_1265-1)del	Turkey	Yes	F	13	18
n.a.	n.a.	n.a.	n.a.	[22]	10–11	not defined	not defined	India	Yes	F	21	19

* Combined heterozygous to c.-20A>G; ^1^ Alu-mediated deletion; ^2^ TSS_putative transcription start site; ^3^ diagnostic primer sequence designed for this study; n.a., not available.

## Data Availability

The authors confirm that the data supporting the findings of this study are available within the article.

## Abbreviations

The following abbreviations are used in this manuscript:

*FERMT1*	Fermitin Family Member 1
bp	Base Pair
cDNA	Complementary DNA
Co	Control
Del	Deletion
DMEM	Dulbecco’s Modified Eagle Medium
EB	Epidermolysis Bullosa
F	Father
*GAPDH*	Glycerinaldehyd-3-phosphat-Dehydrogenase
GRCh37	Genome Reference Consortium Human Build 37
HGMD	Human Genome Mutation Database
IGV	Integrative Genome Viewer
Ins	Insertion
JEB	Junctional Epidermolysis bullosa
kb	Kilo base
KEB	Kindler Epidermolysis bullosa
*KIND1*	Kindlin 1
*LAMB3*	Laminin, beta 3
M	Mother
mRNA	Messenger Ribonucleic Acid
NGS	Next Generation Sequencing
OMIM	Online Mendelian Inheritance in Man
P	Patient
qPCR	Quantitative Polymerase Chain Reaction
RT	Revere Transcription
UV-B	Ultraviolet-B
